# Non-protected areas demanding equitable conservation strategies as of protected areas in the Central Himalayan region

**DOI:** 10.1371/journal.pone.0255082

**Published:** 2021-08-05

**Authors:** Avantika Thapa, Pujan Kumar Pradhan, Bheem Dutt Joshi, Tanoy Mukherjee, Mukesh Thakur, Kailash Chandra, Lalit Kumar Sharma

**Affiliations:** 1 Zoological Survey of India, Kolkata, India; 2 University of Calcutta, Kolkata, India; Amity University, INDIA

## Abstract

The present study aims to explore the mammalian diversity of Darjeeling district using camera traps along with questionnaire survey in protected area (PA) and non- protected area (Non-PA). We also attempted to understand the influence of habitat variables on mammalian species richness using the generalized linear mixed models (GLMM). A total of 30 mammal species were recorded of which 21 species were detected through camera trapping with the most abundant records of barking deer (*Muntiacus muntjak*) and least of the elusive Chinese pangolin (*Manis pentadactyla*) and red panda (*Ailurus fulgens*). Additionally, melanistic forms of four mammals were also recorded. The mammalian species richness, their capture rate and naïve occupancy did not differ significantly among the PA and Non-PA. The GLMM revealed that the proportions of oak and bamboo in the forest, percentage canopy cover and camera trap operational days (*w*AICc = 0.145, *w*BIC = 0.603) were significant predictors of species richness in the study. We suggest Non-PA forest of Darjeeling should be given equal conservation importance as to the PA. Landscape based conservation planning will be imperative for achieving long term conservation goals in the study area.

## Introduction

Globally regions with high biological diversity such as the Himalayas are poorly explored [1] because of rugged terrain and logistics [2, 3]. These areas are now getting vulnerable because of increasing anthropogenic pressures, land use change [4] and climate change [5, 6]. Developing informed conservation and management plans for these hotspots requires vital information on species, like presence absence, occupancy and population [7]. Hence, enumerating the vertebrate diversity of an area is the first step to know about existing fauna and the interaction of these species with the environment [8, 9]. However, systematic data on majority of the species remains scanty in the Himalayan region [1, 10]. Limited efforts have been made to explore and systematically monitor animal populations here [11]. The Central and Eastern Himalayan region, richest among all the Himalayan biotic provinces is still not explored sufficiently [12]. Ecosystems in Central Himalayas are getting altered enormously in unprecedented rate to meet the needs of the growing human populations at spatial scale threatening several species [13]. In the Central Himalayan biotic province, Darjeeling district has unique environmental eco-perception and is home to various endangered mammals and birds [14]. Most of the studies conducted in this landscape are either species specific or limited to documenting biodiversity of PAs only [15, 16].

Protected Areas (PA) are identified as biodiversity rich areas and thus are given special attention with more efforts towards conservation and management [17]. A number of studies have therefore highlighted the imperatives of PA in preventing threatened species from extinction risk [18, 19]. However, forested habitats outside of these PAs are not given importance, because of the misnomer that these areas are not rich in biodiversity and are therefore subject to insufficient monitoring and management policies [20, 21]. In India, PAs are monitored on timescale basis to understand their effectiveness in conserving biodiversity [22] but the non-protected (Non-PA) forests or the territorial forests are managed under the working plans with the main objective of production forestry [23]. Therefore, Non-PAs do not have enough remedies and strategies to conserve wildlife species; rather attention is given to scientific ways of enhancing production of timer and carbon sequestration [24]. These Non-PAs house a good number of species which are ecologically generalist [25] and possess good amount of behavioural plasticity [26]. Here controlled extraction of forest products are permitted to the local residents inhabiting small villages at forest fringes for domestic purpose.

Therefore, present study was designed to assess the mammalian diversity by adopting landscape approach covering PA and Non-PA systematically. Further we compared the mammalian diversity of PAs with the non-PA to test whether the diversity and abundance of mammals is different in two types of forests managed under two different regimes. We also aimed at identifying the drivers of mammalian species richness through camera trapping, sign survey and questionnaire survey.

## Material and methods

### Ethical statement

For conducting the research the research permission was issues by the Principal Chief Conservator of Forest and Chief Wildlife Warden, Government of West Bengal with letter no. 1689/WL/2M-126/2018 dated 05/07/2018. Although no animal handling is required in the present study and we have used only camera trap data which is non-invasively collected. Hence, ethical clearance is not required for the present study. Moreover, informed consent was taken from the questionnaire respondents of the survey for their participation in the study.

### Study area

**Fig 1:**
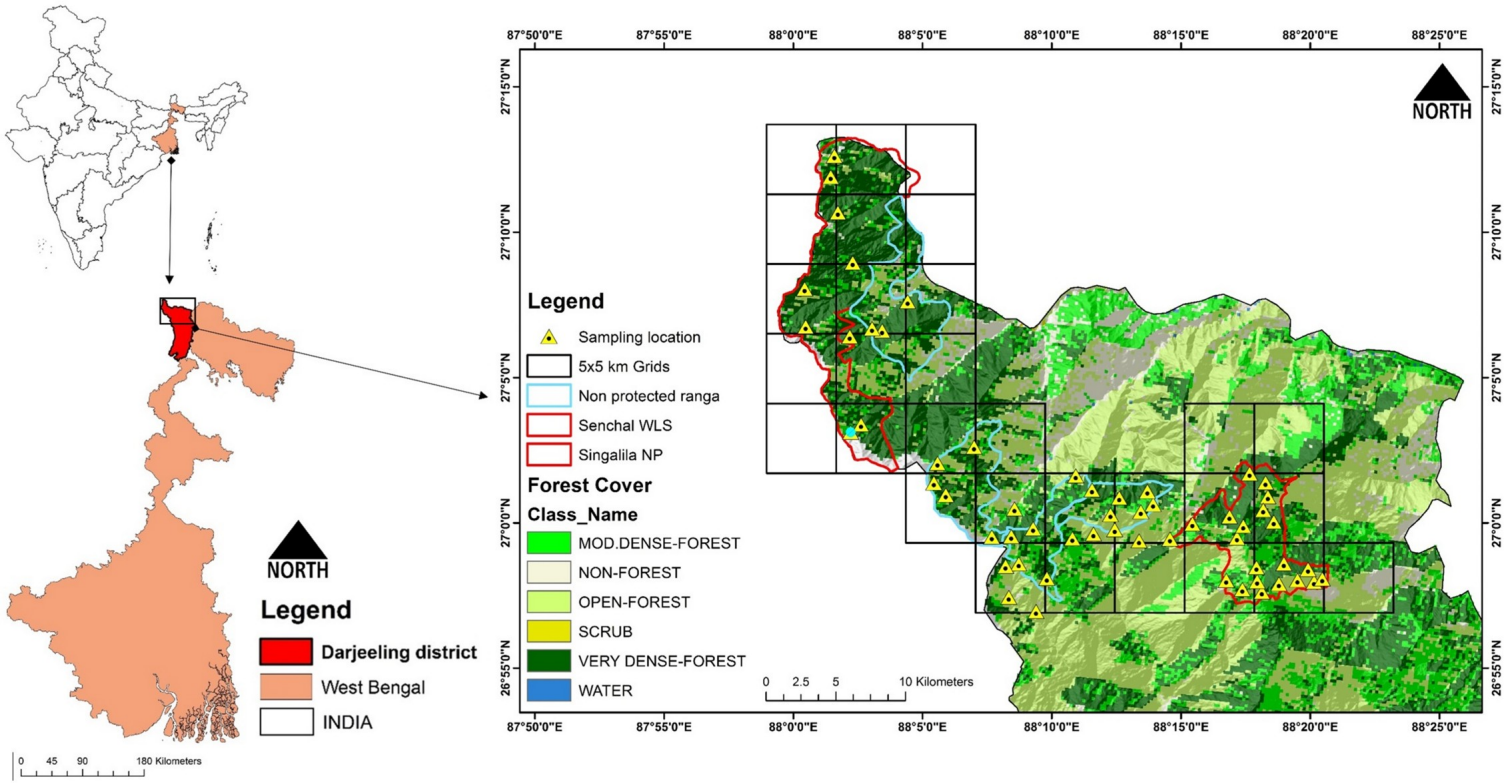
Study area map depicting camera trap locations in protected and non-protected forest ranges of Darjeeling district, West Bengal. WLS, Wildlife Sanctuary; NP, National Park; Non-PAs, Non Protected areas. (Maps are generated using ArcGIS 10.6 www.esri.com).

The study was conducted in Darjeeling district, the northern most district of the state of West Bengal, India, which is also a part of the Central Himalayan Hotspot mostly mentioned in conjugation with the adjacent state of Sikkim as “Sikkim-Darjeeling Himalayas” [27] (Fig 1). This landscape is a part of the Lesser Himalayan ranges which lies between the north-eastern state, Sikkim and adjoining country Nepal with elevation ranging from 150 to 3700m [28]. Darjeeling district has high human population densities [29], resulting into elevated anthropogenic pressure on the wildlife habitats; the landscape is also impacted by the high influx of tourist [29].

Although the Darjeeling district has four PAs *viz*., Singalila National Park (SNP), Senchal Wildlife Sanctuary (SWLS), Mahananda Wildlife Sanctuary and Jorepokhri Salamander Sanctuary but the district also has good quality forest outside the PA network which is managed under Darjeeling Territorial Forest Division. The forests of PAs and Non-PAs possess similar types of forest types *i*.*e*., tropical, temperate and alpine forest. The dominant species of trees in the tropical forest (up to 1200m) includes *Schima wallichii*, *Gmelina arborea*, and *Litsea cubeba*. *Alnus nepalensis*, *Exbucklandia populnea*, and *Quercus lamellose* are the dominant tree species in the temperate forest type (up to 3000 m). The Alpine forest (up to 3636m) comprises few species such as *Abies densa* and *Juniperus pseudosabina* with bushy vegetation like *Gaultheria sp*., *Carex inclinis*, and *Allium wallichii*. A large part of the Non-PAs consist of historical plantations of non-native trees such as *Cryptomeria japonica*. The Non-PAs in the study area are present in mosaic with human settlement and anastomosed by metaled or non-metaled roads. Residents of villages on the fringes of these forests collect fire wood, fodder, bamboo, leaf litter and other non-timber forest products for domestic purpose from here. The study landscape is home to some of the endangered species such as Chinese pangolin (*Manis pentadactlya*), Red panda (*Ailurus fulgens*), Clouded leopard (*Neofelis nebulosa*), Asiatic black bear (*Ursus thibetanus*), and Common leopard (*Panthera pardus*).

### Data collection

For documenting of mammalian species in the study landscape we deployed camera traps inside the forest and conducted questionnaire surveys in the forest fringe villages. The sampling efforts were systematically made in two distinct management areas (PA and Non-PA). We sampled four representative ranges of PAs (two each in Singalila NP and Senchal WLS) (Fig 1) and four Non-PA ranges (Darjeeling directorate, Ghoom-Simana, Tonglu and south Rimbick range) for comparative analysis.

We used three types of camera traps *viz*., spypoint force-11D, boly trail game camera and browning trail camera for the study. Forest areas were divided into 5 x 5 km grids and the grid size was fixed considering the behaviour and home range size of the largest mammal [30]. Out of the 21 gird, camera traps were placed in 14 logistically accessible grids in which the number of cameras ranged from three to five. The average distance between the camera traps was two kilometres (Fig 1). To equalize our sampling effort in both the types of ranges were sampled equal number of grids and cameras trap locations. We deployed 60 camera traps, 30 in each PA and Non-PA forest ranges. These cameras were placed on existing game trails, human trails and in open spaces inside the forests. To minimize the false triggering understory growth was selectively cleared (without modifying natural habitat). The cameras were mounted 40–60 cm above ground on tree trunks without lures [31]. During the study duration (01 November, 2018 to 28 February, 2020) we made an effort of 3865 camera trap nights (1935 trap nights in PA and 1930 trap nights in Non-PA). A total of 4730 photographs captured were sorted into folders for each species and tagged with their respective scientific names in software “Digikam” using package “camtrapR” [32] in R studio. We generated data sheet with date and time information of every capture in 30 minutes interval [33]. All camera traps’ images were carefully visualized to identify the species whereas doubtful and unclear image were excluded from the analysis.

In addition to camera trapping, a face-to-face open ended and semi structured questionnaire surveys (n = 334) was conducted in the forest fringe villages (n = 44). A minimum of 30% of the total household in every village was surveyed [34, 35] following the National Sample Survey Organization, Government of India guidelines [36]. The questions were framed to understand the knowledge of the village communities about the presence of mammalian species in the landscape. The respondents were shown photographs of mammals reported to be present in the area based on distribution of the species from IUCN [37]. We asked the respondents about the current trend of animal population in their locality and gathered information on hunting practises.

## Data analysis

The completeness of our sampling effort was tested using rarefaction curve analysis in EstimateS software [38] which uses the abundance matrices at 95% confidence interval [39]. The species accumulation curve was plotted against cumulative camera trap days in both PA and Non-PA. The number of captures of each species on each day was then randomized 1000 times to derive results. The diversity indices of the mammalian species detected in the PA and Non-PA sites based on the data collected was analysed.

For the comparative analysis of the capture rate and naïve occupancy of species in two types of study ranges, we used a nonparametric Kruskal-Wallis test [40]. The species richness was estimated as the number of species appearing in each camera trap station; capture rate was calculated as the total number of captures of a particular species divided by the operational period of a camera trap station [41]. Whereas, the naïve occupancy of each species was calculated as the total number of camera trap locations of a particular species detected divided by the total number of camera trap stations surveyed [42]. The estimates of the naïve occupancy and capture rates for the species with four or less photo captures were not compared and kept only in the enumeration table [43]. Further the difference in the Shannon’s diversity indices between PA and Non-PA was tested using the Hutcheson’s test [44].

### Understanding the influence of habitat variables on the species richness in PA and non-PA ranges

We attempted to understand the influence of habitat variables on mammalian species richness using generalized linear mixed models (GLMM) [45]. The ‘glmer’ function of package “nlme4” [46] in R Studio with Poisson distribution with log link function preferred for count data [47] was used. We used diagnostic plots to validate the distribution of the residuals. A generalized linear mixed-effect model was constructed with management (PA/Non-PA) as a random effect which allows for correction of unequal and within-group errors [48]. Multi-collinearity among variables was checked using Pearson’s correlation test, and the correlated variables (R^2^ ≥ 0.70) were discarded from the analysis [49]. The number of mammalian species in each camera trap station (site) was taken as a response variable [50]. The predictor variables used in this analysis includes the elevation, management area (PA/Non-PA), canopy cover, human captures, distance of the camera trap station from nearest road and village (haversine distance) [51], the proportion of dominant tree species and forest type (S1 Table). The topographic elevations were extracted from SRTM image downloaded from Earth Explorer (http://earthexplorer.usgs.gov/) using 30m spatial resolution. The distances of camera trap station from the nearest roads and villages were calculated using the “add polygon” function in Google Earth. Human disturbance in the form of independent human captures in the camera traps at intervals of 30 minutes were calculated as number of captures divided by the total number of operational camera trap days in a particular site. Other variables such as canopy cover were estimated using densitometer and number of each tree species within a 10m radius of the camera trap station were noted during field work [52].

We then ran the function ‘glmer’ including all the 16 variables with the response variable. Non-significant variables were removed in a backward stepwise manner to obtain the most parsimonious model [53]. From a set of different competing models, a model assigned with the evidence of lowest information loss (Kullback-Leibler information loss index) was selected [54]. For this purpose Akaike’s information criterion corrected for small sample size (AIC_c_) and Bayesian information criterion (BIC) [56] were calculated for all candidate models using package “AICcmodavg” [55] and “MuMIn” [56] respectively. The AIC_c_ identifies models with minimum K-L information loss [57] while the BIC identifies the probability that a model represents the truth [58]. Furthermore, the AIC_c_ and BIC weights were evaluated to calculate the relative likelihood of candidate models. The values of weights ranged from 0 to 1 indicating no model support to complete model support [54]. Since, the AIC_c_ weights almost always favours models with greater complexity so we also took into consideration the BIC weights in order to identify the most parsimonious models to maximise the prediction accuracy [57, 58].

## Results

### Species richness, capture frequency and site occupancy

A total of 30 species of mammals were recorded during the study (questionnaire and camera trapping) of which 9 species was recorded through questionnaire survey only; two species (marbled cat *Pardofelis marmorata*, golden cat *Catopuma temminckii)* through camera trapping only and 19 species were detected common to both the methods (Table 1). The camera trap generated 2221 independent detections of 21 mammalian species in total of 3865 trap nights (1930 nights in PA and 1935 nights in Non-PA). The species accumulation curve reached an asymptote in case of both PA and Non-PA approximately around 100 days and 155 days respectively (S1 Fig). All species richness estimators (ACE, Chao 1, ICE, Chao 2, Jacknife 1, Jacknife 2 and Bootstrap) estimated species richness ranged from 16.02–18.33 for PA. For Non-PA the estimates ranged from 15.3–19.55 (S1 Fig). The mean species richness in the PA and Non-PA was found to be 4.78 (±0.30). The Shannon diversity index for PA and Non-PA were 3.30 and 3.27 with 18 and 19 mammalian species detected in the respective sites. The Hutcheson’s t-test score of 0.44 corresponding to *p* = 0.657 indicating that there was no significant difference in mammalian diversity between the two sites.

**Table 1:** A list of mammal species recorded during the study, their functional guild, scientific name, common name, IUCN status, IWPA status, CITES status, capture rate, total number of captures of each species and naïve occupancy in PA and non-PA ranges of Darjeeling district, North Bengal.

Scientific name	Common name	IUCN Status	IWPA Status	CITES Status	Total camera captures		Capture rate		Naïve occupancy	
					Types of Sites		Types of Sites		Types of Sites	
					PA	Non-PA	PA	Non-PA	PA	Non-PA
*Panthera pardus*	Common Leopard*^,^^#^	VU	I	I	56	54	0.032 (±0.009)	0.038 (±0.011)	0.467	0.633
*Prionailurus bengalensis*	Leopard cat *^,^^#^	LC	I	II	77	45	0.028 (±0.009)	1.667 (±0.016)	0.467	0.367
*Pardofelis marmorata*	Marbled cat*	NT	I	I	-	9	-	0.033 (±0.016)	-	0.300
*Catopuma temminckii*	Golden cat*	NT	I	I	1	6	0.002	0.004 (±0.002)	0.033	0.167
*Vulpes vulpes*	Red Fox^#^	LC	II	-	-	-	-	-	-	-
*Lutra sp*.	Otter^#^	-	II	-	-	-	-	-	-	-
*Neofelis nebulosa*	Clouded Leopard^#^	VU	I	I	-	-	-	-	-	-
*Herpestes edwardsii*	Indian Grey Mongoose*^,^^#^	LC	II	-	-	1	-	0.001	-	0.033
*Mustela kathiah*	Yellow-bellied weasel*^,^^#^	LC	II	-	2	-	0.0005		0.033	-
*Cuon alpinus*	Dhole^#^	EN	II	-		-	-	-	-	
*Capricornis milneedwardsii*	Mainland serow*^#^	VU	I	I	47	21	0.025 (±0.007)	0.003 (±0.002)	0.467	0.167
*Naemorhedus goral*	Himalayan goral*^,^^#^	NT	III	I	8	-	0.005 (±0.004)	-	0.100	-
*Muntiacus vaginalis*	Barking deer*^,^^#^	LC	III	-	512	383	0.357 (±0.078)	0.199 (±0.051)	0.833	0.733
*Lepus nigricollis*	Indian hare*^,^^#^	LC	IV	_	1	75	0.001	0.041 (±0.016)	0.033	0.200
*Ochotona thibetana*	Moupin Pika^#^	LC	-	-	-	-	-	-	-	-
*Bos gaurus*	Gaur^#^	VU	I	I	-	-	-	-	-	-
*Hemitragus jemlahicus*	Himalayan Tahr^#^	NT	I	-	-	-	-	-	-	-
*Viverra zibetha*	Large Indian civet *^,^^#^	LC	II	III	166	35	0.051 (±0.03)	0.0.016(±0.006)	0.267	0.300
*Paguma larvata*	Masked Palm Civet *^,^^#^	LC	II	III	16	13	0.01 (±0.004)	0.016 (±0.008)	0.233	0.200
*Ursus thibetanus*	Asiatic black bear *^,^^#^	VU	II	I	28	1	0.008 (±0.004)	0.0001	0.167	0.033
*Hystrix brachyura*	Himalayan crestless porcupine*^,^^#^	LC	II	-	14	24	0.002 (±0.001)	0.02 (±0.015)	0.167	0.200
*Ailurus fulgens*	Red Panda *^,^^#^	EN	I	I	4	1	0.002 (±0.001)	0.0004	0.100	0.033
*Sus scrofa*	Wild Boar *^,^^#^	LC	III	-	277	115	0.122 (±0.035)	0.052 (±0.015)	0.700	0.667
*Callosciurus pygerythrus*	Hoary-bellied Himalayan Squirrel*^,^^#^	LC	II	-	41	4	0.014 (±0.006)	0.004 (±0.002)	0.200	0.133
*Martes flavigula*	Yellow-throated Marten*^,^^#^	LC	II	III	12	12	0.004 (±0.002)	0.007 (±0.003)	0.233	0.267
*Macaca assamensis*	Assam Macaque*^,^^#^	LC	II	II	5	5	0.011 (±0.008)	0.003 (±0.002)	0.100	0.100
*Mus sp*.	Mouse*^,^^#^	LC	-	-	96	53	0.031 (±0.02)	0.03 (±0.02)	0.233	1.767
*Belomys pearsonii*	Hairy-footed Flying Squirrel^#^	LC	II	_	-	-	-	-	-	-
*Arctictis binturong*	Binturong^#^	VU	I	III	-	-	-	-	-	-
*Manis pentadactyla*	Chinese Pangolin*^,^^#^	CR	I	_	-	1	-	0.0002	-	0.133

Note

*species captured through camera traps

^#^species confirmed through questionnaire survey; “IUCN” International Union for Conservation of Nature, “IWPA” Wildlife (Protection) Act, 1972 “CITES” Convention on International Trade of Endangered species. “PA” Protected Areas “Non-PA” Non Protected Area.

**Fig 2:**
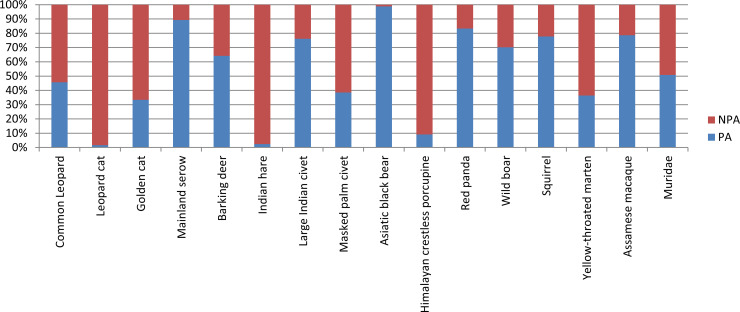
Proportion of naïve occupancy of species in protected area and non-protected area forest ranges of Darjeeling district, West Bengal.

No significant difference was observed between the mean capture rate of the 16 common species found in PA and Non-PA (Kruskal–Wallis, H = 10.89; *P* = 0.795) (Table 1). Furthermore, the naïve occupancy of each of these species did not vary significantly between PA and Non-PA of the study landscape (Kruskal–Wallis, H = 0.42; *P* = 0.511; Fig 2).

### Influence of habitat variables on the species richness

**Fig 3:**
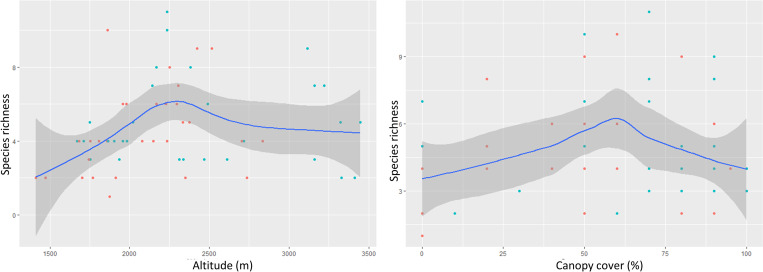
Graph presenting species richness in protected and non-protected forest ranges of Darjeeling district. (a) Species richness maximum at mid elevation of ~2200m (b) Species richness maximum at intermediate cover of ~60% with 95% confidence interval.

The GLMM model revealed that four variables *viz*., proportion of oak trees, proportion of bamboo, percentage of canopy cover and camera trap operational days (*w*AICc = 0.145, *w*BIC = 0.603) were significant predictors of mammalian species richness in the study (Table 2). The number of operational days of a camera trap on a camera station influenced the species richness significantly (*p =* 0.00) in all the top three models (Table 3). Hence, a unit positive change in the number of operational days of camera trap increased the possibility of capturing a new species by 0.005 (β) at a camera trap station. The proportion of oak trees (*p =* 0.008, β = 0.014) and bamboo species (*p =* 0.02, β = 0.006) present in the camera trap stations were also found to influence the species richness positively (Table 3). However, the canopy cover was found to have slight negative association with the species richness (*p =* 0.09, β = -0.135). There was no significant relation of species richness with the camera trap located in PA. Hence, richness did not differ among camera taps located in PA and Non-PA. The species richness increased with altitude and maximized at ~2200 m then decreased with further rise in elevation (Fig 3A). The same pattern of maximum richness in the intermediate area was shown by canopy cover as well; it produced a hump at ~60% then gradually decreased with increasing canopy cover (Fig 3B).

**Table 2:** The three most parsimonious generalized linear mixed-effects models representing the most influencing predictor variables based to *wA*IC and *w*BIC.

Model no.	Model attributes	k	LL	ΔBIC	*w*BIC	ΔAIC_c_	*wA*IC
1	PA + Prop_Oak** + Prop_Bamb* + Canopy_std**˙** + Op_Days*** + Alt_std	8	-117.95	0	0.603	0	0.145
2	PA + HaversineDistR + Prop_Oak* + Prop_Bamb ˙ + Canopy_std + Op_Days***	8	118.51	1.12	0.345	1.1	0.084
3	PA + HaversineDistR + Prop_Oak* + Dist_W_std + Prop_Bamb ˙ + Canopy_std + Op_Days***	9	.118.41	5.02	0.049	1.8	0.059

Note: PA: Protected area; Prop_Oak: Proportion of oak, Prop_Bamb: Proportion of bamboo, Op_Days: Number of operational days, Alt_std: Altitude; HaversineDistR: Distance from Road; Dist_W_std: Distance from Road, K: number of parameters; LL: log likelihood.

**Table 3:** Influence of the predictor variables on mammalian species richness as tested by generalized linear mixed-effects models in Darjeeling, North Bengal.

Response variable	Predictor variables	*β*	SE	z-test	P	
Species Richness	Protected area	0.770	0.138	1.156	0.247	
	Proportion of oak	0.014	0.005	2.629	**0.008**	**
	Proportion of bamboo	0.006	0.002	2.192	**0.028**	*
	Canopy cover	-0.135	0.200	-1.686	**0.097**	.
	Number of operational days	0.005	0.001	4.922	**0.000**	***
	Altitude	-0.086	0.819	-1.061	0.288	
**AIC**	251.9					
**BIC**	268.6					

Significance codes: 0 ‘***’ 0.001 ‘**’ 0.01 ‘*’ 0.05 ‘.’ 0.1 ‘‘ 1.

Out of the 30 species, 16 (53.33%) were found to be common in both the sites (S2 Fig). The naïve occupancy calculated for 30 mammalian species were ranging from 0.03–0.83 (Table 1). In addition to this, different body morphs of four mammalian species were also captured, in which the melanistic morphs were the most prevalent (S3 Fig). The black morphs of barking deer (n = 4) and common leopard (n = 5) were captured in PA and as well as Non-PA. A single captures of melanistic golden cat and leopard cat were captured only from Non-PA forest range. The captures of these melanistic forms of the three animal’s *viz*., barking deer, common leopard and golden cat were captured from the same camera trap station placed in Non-PA.

Questionnaire survey (n = 334) revealed the presence of nine more species *viz*., red fox (*Vulpes Vulpes*), otter *(Lutra sp)*.,hairy-footed flying squirrel (*Belomys pearsonii*), binturong (*Arctictis binturong*), clouded leopard (*Neofelis nebulosi*), moupin pika (*Ochotona thibetana*), dhole (*Cuon alpinus*), gaur (*Bos gaurus*) and Himalayan thar (*Hemitragus jemlahicus*)which were not captured during the study period (Table 1). About 91% of the respondent showed positive attitudes towards the wildlife conservation (S4 Fig).

## Discussion

### Comparative analysis of mammalian richness in PA and Non-PA ranges

The forest habitat of Darjeeling is home to several species of mammals and other faunal elements [14, 59]. However, no systematic account was available on mammalian diversity from the landscape. Hence, the present study is the first of its kind which has attempted to record the mammalian fauna in the hills of Darjeeling through systematic field work. The results indicate that there are about 30 species of mammals present in the landscape (Table 1). We recorded first photographic evidences for the presence of elusive cats like marbled cat and golden cat.

Further, through the present study we documented that the diversity and richness of mammals did not varied significantly between two types of ranges i.e., forest ranges of PA and Non-PA. There is also a misnomer, that forest areas of PAs are rich in mammalian diversity in comparison to Non-PA because of protection of PA by law and enforcement [24]. However, the present study revealed that there was no significant difference in the mammalian species richness between the PA and Non-PA, inferring need of adopting scientific monitoring and management strategies in Non-PA as well. Sixteen out of the 21 mammals encountered through camera trapping were common to both the sites. In fact, the Non-PA forest ranges provide habitat for more number of threatened species than the PA ranges (Table 1), like the Chinese pangolin (CR) reported only in Non-PA forest. Similar results were also observed in Bhutan where both the Chinese and Indian pangolin was only reported from Non-PA forest ranges [10]. The presence of conservation priority species such as Chinese pangolin only in Non-PA indicates that these habitats require urgent conservation strategy to safeguard the most trafficked animal on the globe [60].

Moreover, the present comparative analysis of the naïve occupancy and capture rates of the mammals in PA and Non-PA did not differed significantly. The capture rates are considered useful proxies in understanding the site occupancy and relative abundance of species [61]. Few other studies have documented significant differences in the species richness between the PAs and Non-PAs [10, 62]. However, no significant difference of mammalian richness among PA and Non-PA in the present study could be corroborated with the fact that during the study period we did not recorded any hunting activity and the forests outside the PA network are of good quality and intact. This is also supported through questionnaire data analysis where 91% respondents showed positive attitude towards wildlife conservation and were interested in conservation (S4 Fig). Moreover, the human photo captures or disturbance has also not impacted the species richness in both PA and Non PAs (Table 3).

### Habitat predictors of mammalian richness

The results of the best GLMM model suggested that the number of species detected may increase on localized sites if the camera traps are kept open for few additional days (Table 2). Additionally, oak and bamboo proportions in camera trap sites were positively associated with the species richness. This can be corroborated with the fact that oak makes up a large proportion of desirable food for variety of vertebrate species with high energy food resource [52, 63, 64]. Nonetheless, the bamboo forests are vital for the survival of species such as red panda and many other species. Moreover, a negative relationship between bamboo forests on mammalian species richness [65, 66] is opposed by our findings.

Further, no significant association of human captures with mammalian richness in the study landscape indicates that the species are not getting disturbed with human presence, or the human presence is limited in the study sites because of rugged terrain and friendly attitude of communities towards wildlife in the landscape. A number of studies are available indicating both positive [67] and negative impact on the species richness [19].

## Conclusion

The Non-PA of the study area is home to 19 mammalian species which includes species of global importance (seven) therefore, conservation strategies must concentrate on increasing the physical connectivity of these areas [68]. These forests ranges with rich biodiversity should be managed in a more scientific manner to sustain and maintain the long term viable mammalian populations. Hence, a landscape strategy should be adopted for the conservation of wildlife and management of land resources in the study area.

## Supporting information

S1 Appendix Questionnaire sheet. (DOCX)

S1 Fig Species accumulation curve showing estimates of six best performing estimators for PA and NPA showing that asymptote was achieved in both the sites. (DOCX)

S2 Fig Venn diagram showing the 16 common species between PA and Non-PA along with two unique species each in each site. (DOCX)

S3 Fig Camera trap images of different types of morphs of four species and first photographic evidences. A) Melanistic *Panthera pardus B)* Melanistic *Prionailurus bengalensis C)* Melanistic *Muntiacus muntjak D)* Melanistic *Catopuma temminckii E) Catopuma temminckii* with rosettes F) *Pardofelis marmorata*.
(DOCX)

S4 Fig Percentage of respondents’ attitude towards wildlife. (DOCX)

S1 Table Habitat covariates used for the Generalized Linear Mixed-effect Modelling (GLMM) of species richness in the study area. (DOCX)
