# Supplementary material for: Non-protected areas demanding equitable conservation strategies as of protected areas in the Central Himalayan region
Source: PLoS One. 2021 Aug 5;16(8):e0255082. doi: 10.1371/journal.pone.0255082 (PMC8341489; doi:10.1371/journal.pone.0255082)
Supplement: S1 Appendix — (DOCX) [file pone.0255082.s001.docx]

**S1 Appendix. Questionnaire sheet.**

**QUESTIONNAIRE SURVEY**

**SOCIO-ECONOMIC, PERCEPTION OF WILDLIFE**

Name of the village___________________ GPS Coordinates ___________N___________S

Division: ______________________________ Range: ______________

**Name of interviewee** ________________________________________________

**1**. (i) Age _______ (ii) gender: (a) Male (b) Female

(iii) Number of persons in household (gender wise adult and kids) _________________

**2. What is your educational level?**

(a ) Illiterate ( b) Primary school certificate ( c) Middle school certificate (d ) High school certificate ( e) Intermediate or post high school diploma (f ) Graduate or post graduate

(g) Professional or honours

**3. Occupation of head of the Family?**

(a) Unemployed (b) Unskilled worker (c) Semi- field worker (d) Skilled worker (e) Clerical-soft honour (f) Semi- professional (h) Professional

**4. Socio-Economic Class: (Caste Status)_____________________________**

**5. What type of house do you have?**

(a) Pucca (b) Semi-Pucca (c) Kutcha (d) Bamboo/Wood

**6. Do you have electricity connection?** (a) Yes (b) No

**7. What is your source of drinking water?** (a) Tap (b) Stream/river (c) Others

**8. What source of fuel do you use for cooking purpose?**

(a) Fuel wood (b) LPG (c) Kerosene (d) Bio-gas

**9. How long have you lived in this place? And where have you come from? _________**

**10. What is/are your source/s of livelihood? (List in terms of priority if more than one):**

(a) Service (government/private) (b) Agriculture (c) Livestock farming (d) Daily wages/Tea estate worker (e) Hotel/Tourism (e) Others

**11. Do you own livestock?** (a) Yes (b) No

**If yes then mention the No**

(a) Cow: __________ (b) Buffalo: ________ (c) Goats: ________ (d) Sheep: _________ (e) Poultry: ________ (f) Pig: _________

**12. Purpose of Keeping Livestock:** (a) Selling meat/milk (b) agriculture (c) personal consumption

**13. Grazing in forest: ____________________**

14. Animal diseases. Foot/Mouth _____________________

**15. How many live stocks are died due to disease? __________________**

**16. Do you own agricultural land?** (a) Yes (b) No

**17. What is the size of your land holding? (bighas/kathas) __________**

**SECTION 2: PERCEPTIONS ABOUT WILDLIFE**

**1) Infringement area Yes/No:**

**2) Are you facing wild animals problems :**(a) Yes (b) No

**3) Problems from Animals**

i. Which animals come to this village:

ii. How many animals visiting in the village:

iii. Timing of raiding behavior Diurnal / Nocturnal

iv. Frequency of raiding (Daily, Weekly, Month, Year)

**4) Does single individual or herd visiting here.______________________**

**5) Type of Conflict?**

I. Crop damage ii. Human attack iii. Livestock attack

Timing of the conflict: __________

**6) Collect some historical data?**

i. How long the animals come to this area________________

ii. Present status of the animal in this area________________

iii. How often the animals come to the village _____________

iv. Which season they arrive most? ______________________

**7) Main Prey species of carnivores animal:-__________,__________,____________,_____________,_______________,_________.**

**8) Cause of declination of prey species:**

**________________________________________________________________________**

**9) Cause of degradation of ideal habitat of animals:**

**________________________________________________________________________**

**10) Do you know about the threatened vertebrates or animals of India/ state?**

**___________,____________,____________,_____________,_____________,___________**

**11) Do you know about any wildlife protections acts and rules of the country or the state?**

**___________________________________________________________________________**

**12) Why do animals need protection?**

**_____________________________________________________________________________**

**13) What is the role of carnivore/ herbivore/ Birds/ herpato fauna in the wild?**

(a) To control prey population/ to keep the ecological balance

(b) To destroy, subdue or eat other animals/

(c) To destroy forests & damage agriculture/food resources/human habitation

(d) Other (specify)

(e) None

(f) Do not know

**14) Why do you think Wild animals stray into human habitation?**

(i) Loss/Difficult of/in capturing natural prey (ii) Easy food i.e. Livestock, crops

(iii) Natural Calamities (iv) Negotiate the forest block

(v) Encroachment & Fragmentation of forested habitat (vi) By chance (vii) migratory corridors (viii) Food attractant (ix) to give birth to litter (x) others

**15) Do you practice some form of ritual to ward off animal attacks? If yes, is it effective?**

__________________________________________________________________________

**16) Ethno zoological knowledge holds by the interviewee? Which animal and their parts are frequently used by local people for**

(a) Religious (b) Medicinal purpose (c) Food

(d) Ornamental/Economical (e) Others

**___________________________________________________________________________________________________________________________________________________**

**17) Do you have secret groves/believe/practices in the village?**

**___________________________________________________________________________**

**18) Are aware about the compensation payable at forest department? Yes/No**

i. If yes, there are you happy with this (Yes/No)

ii. If no- Please suggests what kind of alternate you need?

**19) What does forest department do in HWC mitigation and how effective the methods are?**

**20) What crop protection measures are being used by you Yes / No/**

**21) Please, indicate your attitude toward the following:** {Like/ Dislike/ Do not know}: (a) Black bear___ (b) Leopard/ Lesser Cat___ ( c) Elephant___ ( d) Deer___ ( e) Nature/ other____

**Comments:**
