# Supplementary material for: Non-protected areas demanding equitable conservation strategies as of protected areas in the Central Himalayan region
Source: PLoS One. 2021 Aug 5;16(8):e0255082. doi: 10.1371/journal.pone.0255082 (PMC8341489; doi:10.1371/journal.pone.0255082)

**S1 Fig. Species accumulation curve showing estimates of six best performing estimators for PA and Non-PA showing that asymptote was achieved in both the sites.**


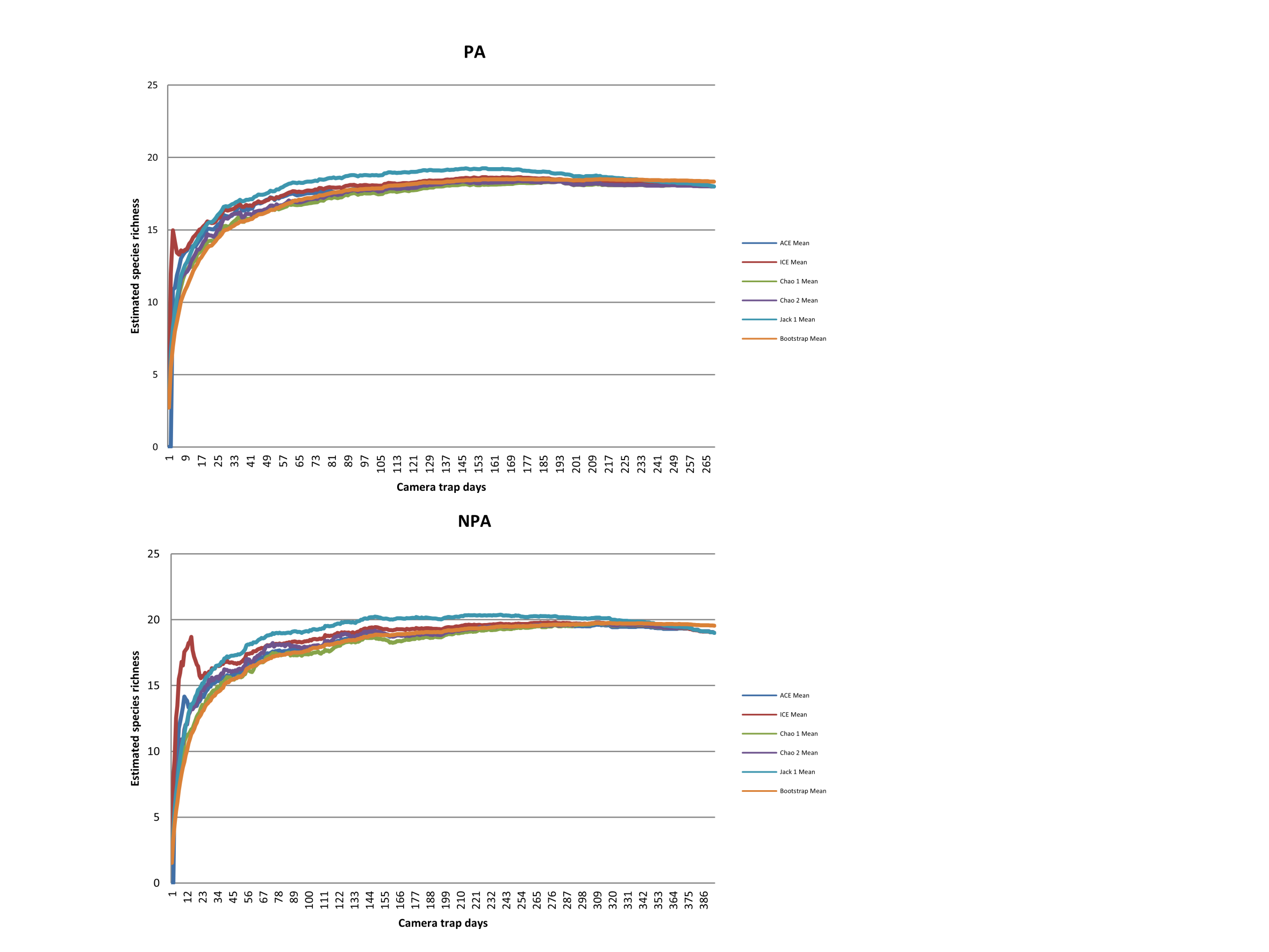

Supplement: S1 Fig — (DOCX) [file pone.0255082.s002.docx]
