# Supplementary material for: Non-protected areas demanding equitable conservation strategies as of protected areas in the Central Himalayan region
Source: PLoS One. 2021 Aug 5;16(8):e0255082. doi: 10.1371/journal.pone.0255082 (PMC8341489; doi:10.1371/journal.pone.0255082)

**S2 Fig. Venn diagram showing the 16 common species between PA and Non-PA along with two unique species each in each site.**


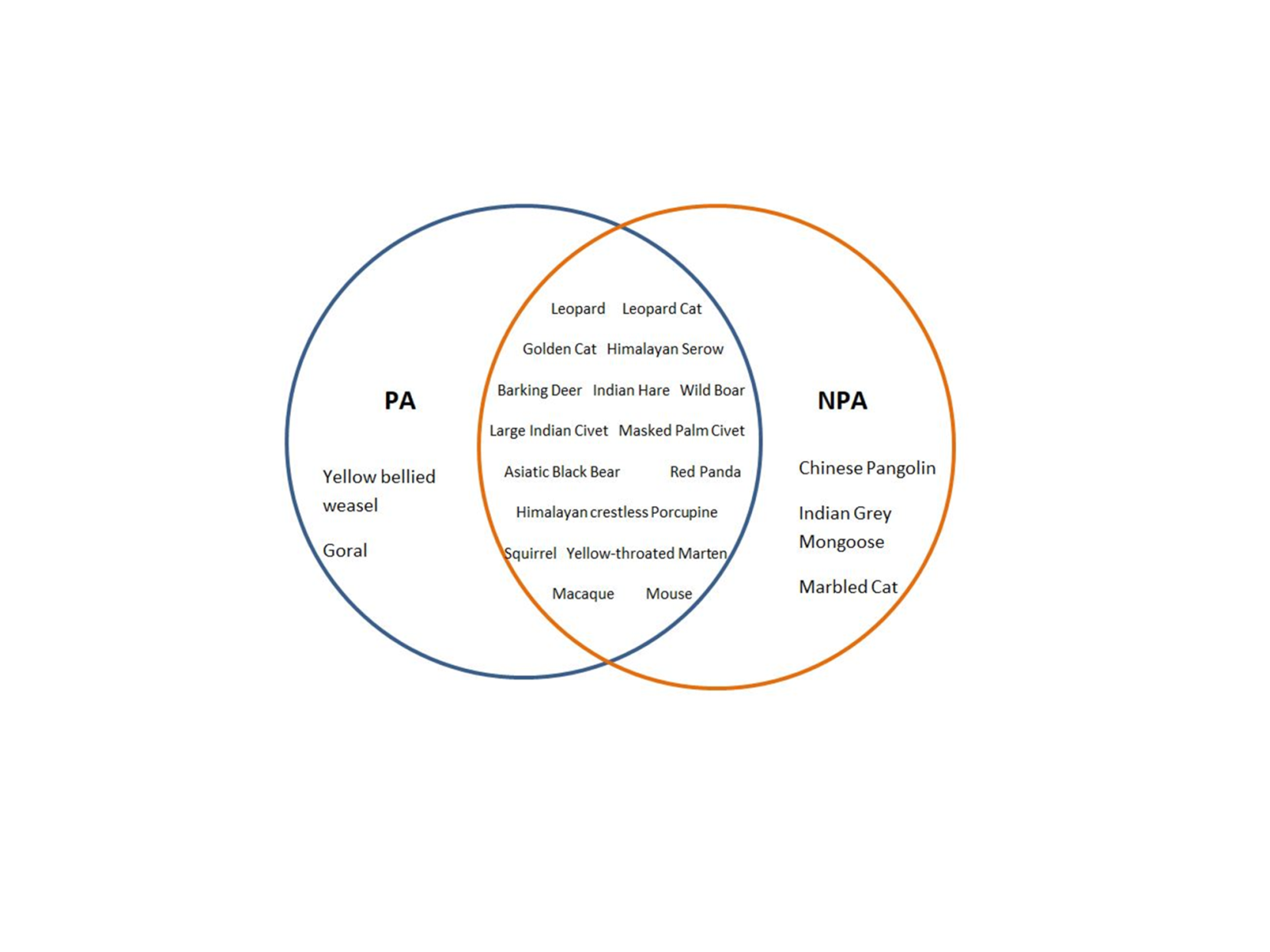

Supplement: S2 Fig — (DOCX) [file pone.0255082.s003.docx]
