# Supplementary material for: Non-protected areas demanding equitable conservation strategies as of protected areas in the Central Himalayan region
Source: PLoS One. 2021 Aug 5;16(8):e0255082. doi: 10.1371/journal.pone.0255082 (PMC8341489; doi:10.1371/journal.pone.0255082)

**S3 Fig. Camera traps images of different types of morphs of four species and first photographic evidences. A) Melanistic *Panthera pardus B)* Melanisti c*Prionailurus bengalensis C)* Melanistic *Muntiacus muntjak D) Catopuma temminckii E) Catopuma temminckii* with rosettes F) *Pardofelis marmorata.***


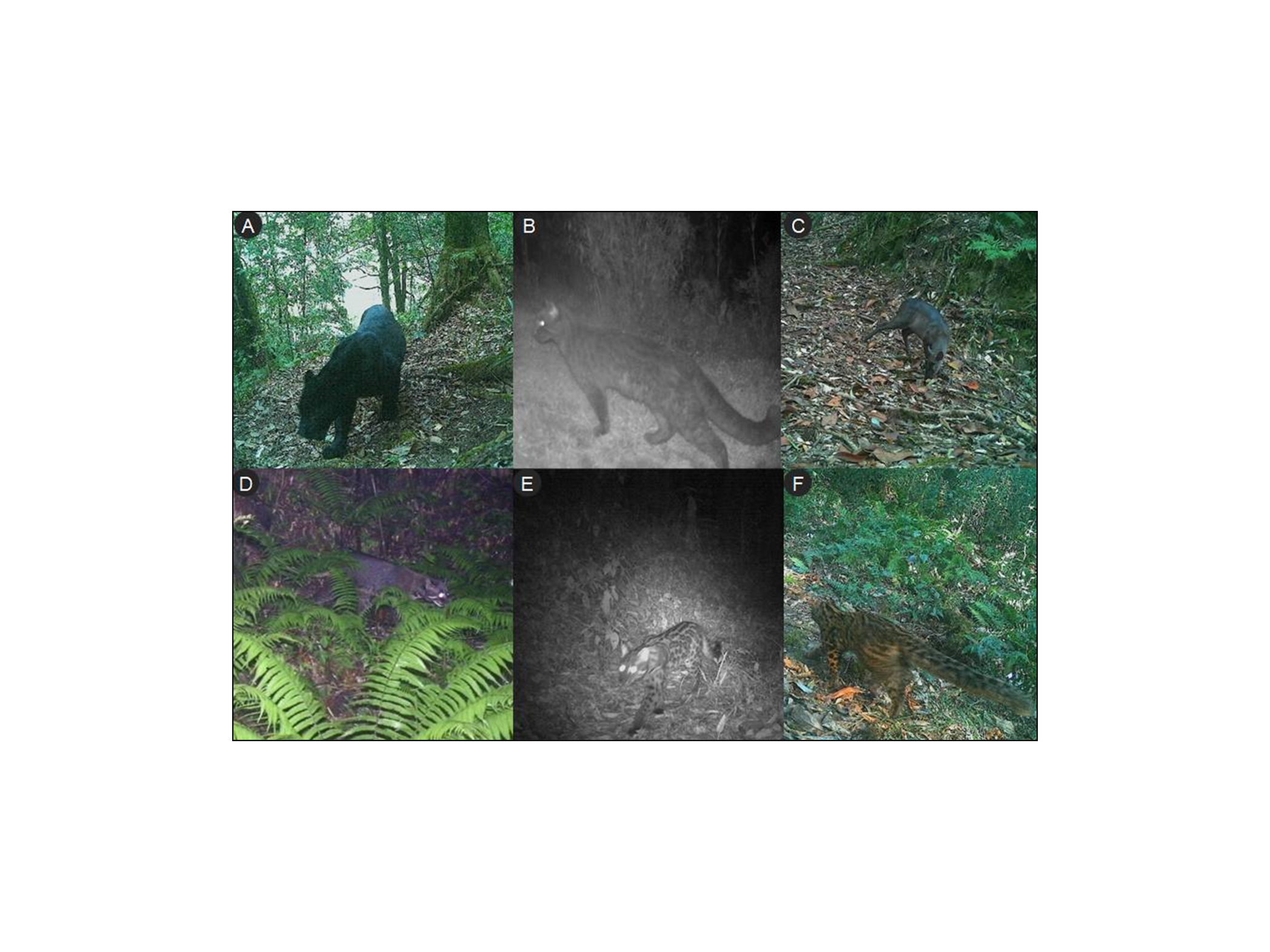

Supplement: S3 Fig — A) Melanistic Panthera pardus B) Melanistic Prionailurus bengalensis C) Melanistic Muntiacus muntjak D) Melanistic Catopuma temminckii E) Catopuma temminckii with rosettes F) Pardofelis marmorata. (DOCX) [file pone.0255082.s004.docx]
