# Supplementary material for: Non-protected areas demanding equitable conservation strategies as of protected areas in the Central Himalayan region
Source: PLoS One. 2021 Aug 5;16(8):e0255082. doi: 10.1371/journal.pone.0255082 (PMC8341489; doi:10.1371/journal.pone.0255082)
Supplement: S4 Fig — (DOCX) [file pone.0255082.s005.docx]

**S4 Fig. Percentage of respondents’ attitude towards wildlife in Darjeeling District.**
