# Supplementary material for: Non-protected areas demanding equitable conservation strategies as of protected areas in the Central Himalayan region
Source: PLoS One. 2021 Aug 5;16(8):e0255082. doi: 10.1371/journal.pone.0255082 (PMC8341489; doi:10.1371/journal.pone.0255082)
Supplement: S1 Table — (DOCX) [file pone.0255082.s006.docx]

**S1 Table: Habitat covariates used for the Generalized Linear Mixed-effect Modelling (GLMM) of species richness in the study area.**

| **Sl. no.** | **Variables** | | **Code** | **Source** |
| --- | --- | --- | --- | --- |
| 1 | Elevation | | Alt_std | SRTM data USGS |
| 2 | Proportion of Bamboo | | Prop_Bamb | Field Observation |
| 3 | Proportion of oak trees | | Prop_Oak |  |
| 4 | Proportion of Pine trees | | Prop_Pine |  |
| 5 | Mixed forest | | Mix |  |
| 6 | Plantation | | PlanT |  |
| 7 | Canopy cover | | Canopy_std |  |
| 8 | Sub-Tropical Vegetation | | STRV |  |
| 9 | Sub-Temperate Vegetation | | STEV |  |
| 10 | Temperate Broad Leaved mixed Deciduous | | TBLD |  |
| 11 | Evergreen Oak Forest | | EOF |  |
| 12 | Hemlock-Rhododendron Forest | | HRF |  |
| 13 | Sub-Alpine Vegetation | | SAV |  |
| 14 | Distance from Water | | Dist_W_std |  |
| 15 | Distance from Road | | HaversineDistR | Calculated using the “add polygon” function in Google Earth |
| **16** | Distance from Village | | HaversineDistV |  |
| **17** | Human Captures | HumanCap | | Capture rate calculated from camera trap data |
| 18 | Operational Days | | Op_Days | Number of days the camera trap was operational in each station |
| 19 | Protection | | PA | Forest department of WB |
